# Supplementary material for: Concentration gradients in evaporating binary droplets probed by spatially resolved Raman and NMR spectroscopy
Source: Proc Natl Acad Sci U S A. 2022 Apr 4;119(15):e2111989119. doi: 10.1073/pnas.2111989119 (PMC9169657; doi:10.1073/pnas.2111989119)
Supplement: Supplementary File [file pnas.2111989119.sapp.pdf]

**Supplementary Information for**

Concentration Gradients in Evaporating Binary Droplets Probed by Spatially Resolved Raman and NMR Spectroscopy

Alena K. Bell[1 †], Jonas Kind[3 †], Maximilian Hartmann[2], Benjamin Kresse[4], Mark V. Höfler[4], Benedikt B. Straub[5], Günter K. Auernhammer[5,6], Michael Vogel[4], Christina M. Thiele[3#], Robert W. Stark[1*]

[1] Physics of Surfaces, Institute of Materials Science, Technical University of Darmstadt, Alarich-Weiss-Str. 16, 64287 Darmstadt, Germany
[2] Institute for Nano- and Microfluidics, Technical University of Darmstadt, Alarich-Weiss-Str. 10, 64287 Darmstadt, Germany
[3] Clemens-Schöpf-Institute for Organic Chemistry and Biochemistry, Technical University of Darmstadt, Alarich-Weiss-Str. 16, 64287 Darmstadt, Germany
[4] Institute of Condensed Matter Physics, Technical University of Darmstadt, Hochschulstr. 6, 64289 Darmstadt, Germany
[5] Max Planck Institute for Polymer Research, Ackermannweg 10, 55128 Mainz, Germany
[6] Leibniz-Institut für Polymerforschung, Hohe Straße 6, 01069 Dresden, Germany

† Both authors contributed equally
# corresponding author, cthiele@thielelab.de
* corresponding author, robert.stark@tu-darmstadt.de

**Supplementary Information**

**Substances**
**Binary Droplets**
Table S1 shows the physical properties of the three substances, namely 1-hexanol, 1-butanol, and 1-butanol-$d_9$. For the experiments pure substances as well as mixtures of protonated 1-butanol and 1-hexanol and protonated 1-hexanol and deuterated 1-butanol-$d_9$ were used.

**Table S1.** Physical properties (refractive index n, boiling temperature T, density ρ, vapor pressure E and surface tension σ) of 1-hexanol, 1-butanol, and 1-butanol-$d_9$. The properties were extracted from the respective material safety data sheets (MSDS).

| | n | $T_b$ (°C) | ρ (g/ml) | E (Pa) at 20 °C | γ (mN/m) at 20 °C | -(∂γ/∂T) (mN/mK) | λ (W/mK) | $c_p$ (J/molK) | *η* (mPa/s) | *D* (m²/s) |
|---|---|---|---|---|---|---|---|---|---|---|
| 1-butanol | 1.399 | 117-118 | 0.81 | $6.67 \cdot 10^2$ | 24.67 | 0.094 | 0.1575 | 176.86 (1) | 2.544 (2) | $0.487 \cdot 10^{-9}$ (3) |
| 1-butanol-$d_9$ | 1.3956 (1-butanol-$d_{10}$) | 116-118 | 0.907 | $5 \cdot 10^2$ | | | | | | |
| 1-hexanol | 1.418 | 156-157 | 0.814 | 93 | 24.91 | | 0.1570 (4) | 243.2 (5) | 0.592 | 0.218 (6) |

**Data analysis**
**Contact Angle Goniometry of Evaporating Droplets**
The shape (contact angle (CA), droplet height (h), wetting radius (r), and droplet radius) of the evaporating droplets was determined as a function of time from laterally acquired contact angle goniometry measurements. Figures S1 and S2 show the CA measurements of all pure substances and the mixtures of 1-butanol and 1-hexanol. The measurements given in figure S1 and figure S2 show non-linear decreases in the height, radius and contact angle of the droplet. As a guide for the eye, straight lines are given in the graph.

A comparison of the wetting radius and height of the 1-butanol-$d_9$/1-hexanol mixture in figure S2 shows that these parameters exhibited opposite non-linear behaviors: the height remained constant while the wetting radius decreased and vice versa. This effect was most prominent at the beginning of the experiment when the light and more volatile 1-butanol-$d_9$ evaporated faster than 1-hexanol. After that, 1-hexanol evaporated more or less like a pure substance. This means that the binary drop evaporated in stick-slip mode, namely, in a mixed mode of constant contact angle (CCA) and constant contact radius (CCR). Figure S2(b) also shows the droplet radius, which is crucial for the lens effect and plays a role in the Raman spectroscopic measurements. It is observed here that the droplet radius and the wetting radius evolved in opposite ways, which is also connected to the stick-slip evaporation mode. In the final state, the wetting and droplet radii, height, and contact angle decreased simultaneously. Such behavior is well known (see, e.g., Park et al. (7)) and has also been observed for pure substances and mixtures of both protonated substances.

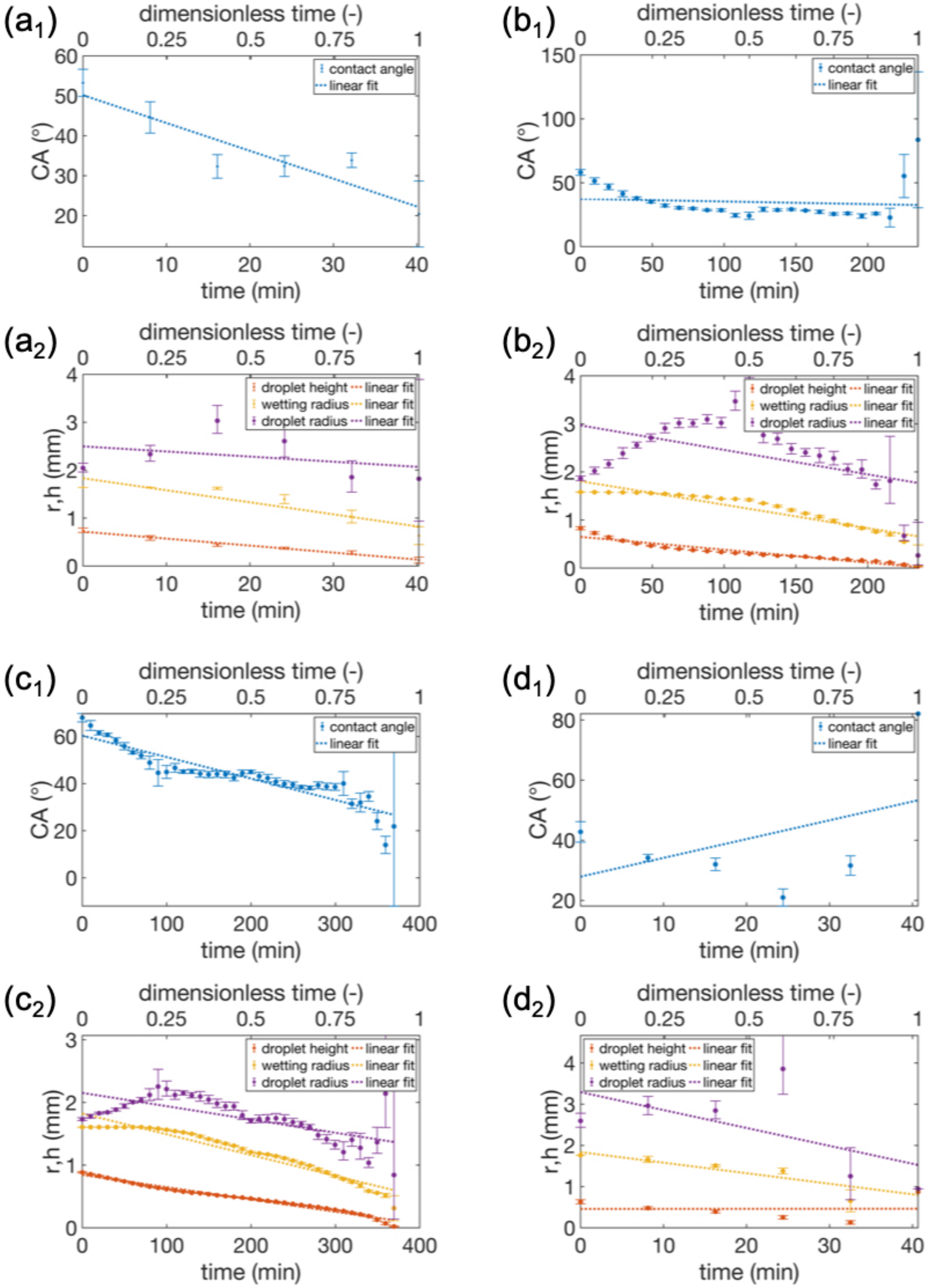


**Figure S1.** Contact angle ($a_1$, $b_1$, $c_1$, $d_1$), wetting radius, droplet radius, and droplet height ($a_2$, $b_2$, $c_2$, $d_2$) of pure 1-butanol (a), a 1-butanol/1-hexanol mixture (b), pure 1-hexanol (c), and pure 1-butanol-$d_9$ (d) droplets. The linear fit is given as a guideline for the eye to observe the wavelike decrease of the different observables easier.

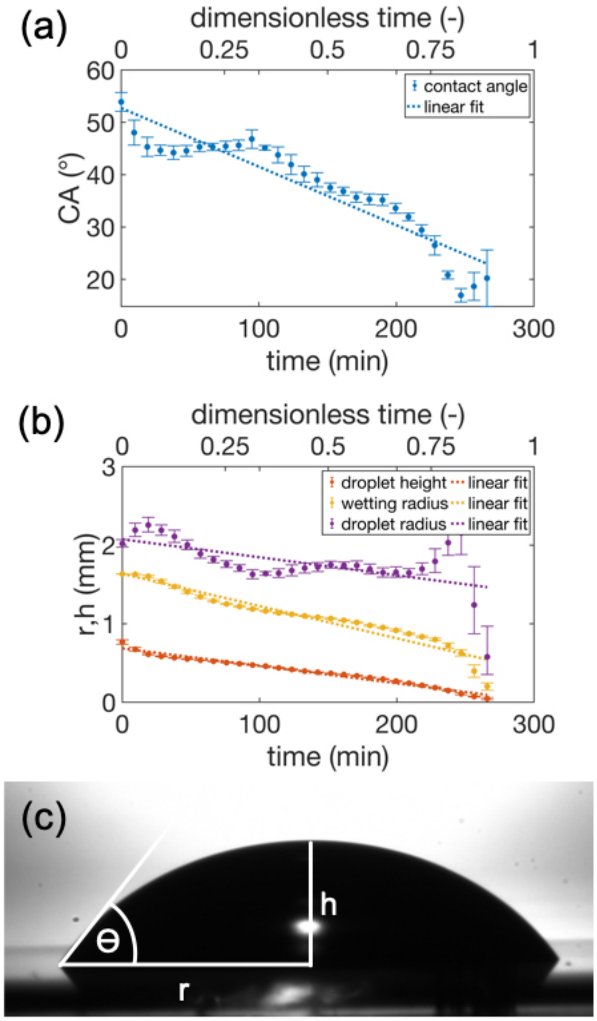


**Figure S2.** (a) Contact angle θ, (b) wetting and droplet radii r, and height h of sessile droplets composed of 1-butanol-*d*₉/1-hexanol. Starting composition was 50:50 mol%. All curves show non-linear behavior, which indicates that the evaporation process occurred in a stick-slip mode. Data for the pure substances are given in figure S1. Straight lines are given in the data plots as a guide for the eye only. (c) Image of the droplet with contact angle θ, droplet height h and contact line radius r indicated.

__Examination of spatially resolved concentration fields with Raman Spectroscopy__

In figure S3, the temporal sequences of depth image scans are given for the pure substances (1-butanol, 1-hexanol, 1-butanol-*d*$_9$) as well as for the protonated 1-butanol/1-hexanol mixture. In figure S3(a-d), the sub images (a) relate to pure 1-butanol, (b) to the mixture of 1-butanol/1-hexanol, (c) to pure 1-hexanol, and (d) to pure 1-butanol-*d*$_9$. In the sub images (e) and (f), the calibration curve and the height of the 1-butanol-*d*$_9$ and 1-hexanol are shown.

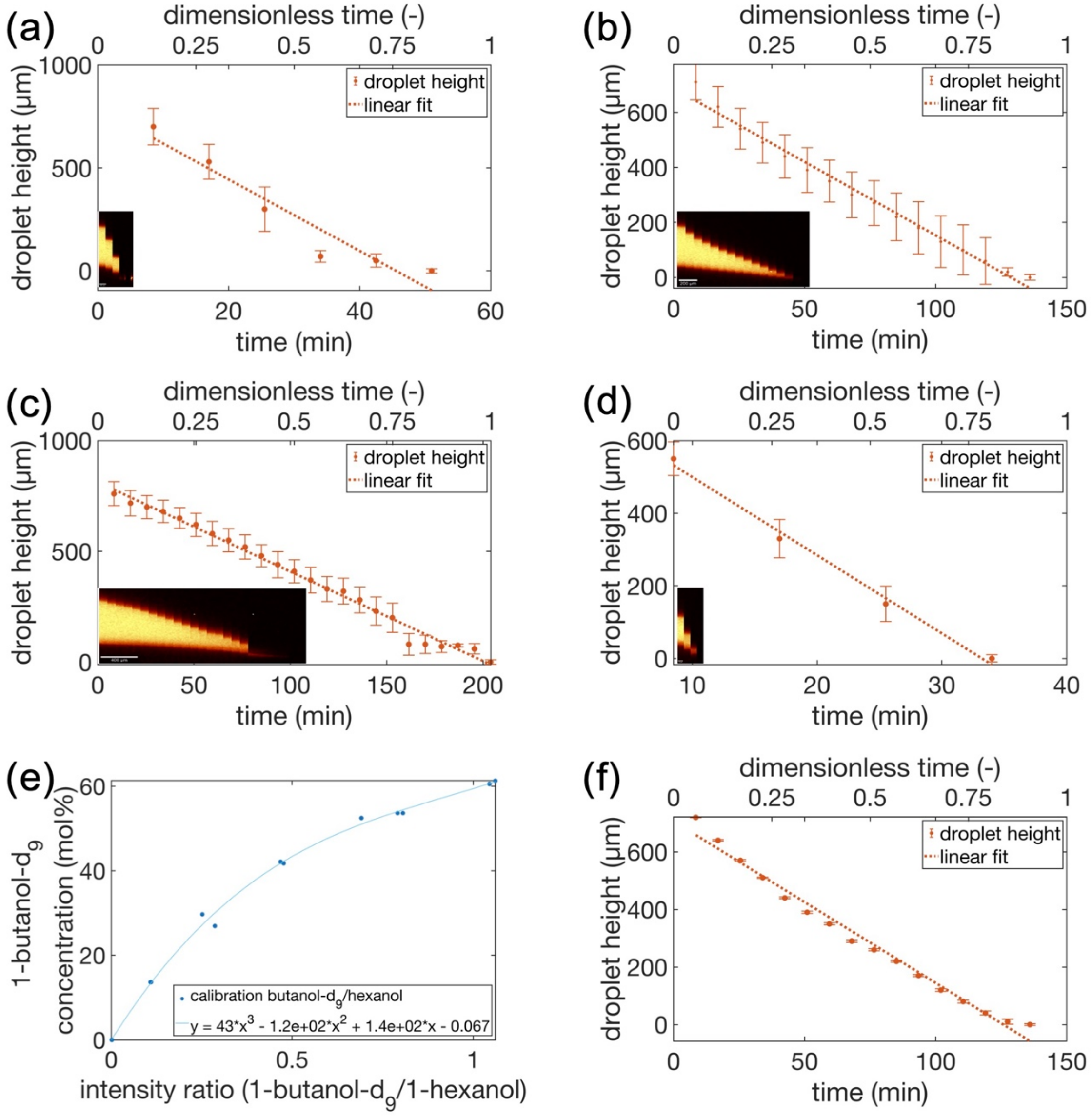


**Figure S3.** Droplet height as a temporal sequence of pure 1-butanol (a), a (protonated) 1-butanol/1-hexanol mixture (b), pure 1-hexanol (c), and pure 1-butanol-*d*$_9$ (d) calculated from Raman spectroscopic image scans as given in the insets of each graph. The inset graphs are taken the same way as in figure 1 (main manuscript) with 90 x 1000 µm$^2$ for each scan, a brighter pixel color corresponds to higher Raman intensity than dark pixel color. Calibration curve for the system 1-butanol-*d*$_9$/1-hexanol fitted with a 3$^{rd}$ order polynomial function (e) and droplet height as temporal sequence of the 1-butanol-*d*$_9$/1-hexanol mixture. The straight line (in a, b, c, d, f) is inserted as a guideline for the eye to observe the wavelike decrease of the droplet height easier.

In figure S3(a,b,c,d,f) a straight line is shown to guide the eye. The slope and wavelike-nature of the decrease of height can clearly be discerned. The droplet height is given over the experimental time for the pure substances and both mixtures with protonated and deuterated 1-butanol and 1-hexanol.

A calibration curve for the system 1-butanol-$d_9$/1-hexanol is given in figure S3(e). In the curve the 1-butanol-$d_9$ concentration is given over the intensity ratio of the integrated intensity of the characteristic peaks of 1-butanol-$d_9$ and 1-hexanol. The characteristic peaks and the filtering parameters are the same ones as for the filtering of the 2D Raman scans given in figures 1 and S3 (insets in the respective graphs). For the calibration curve, Raman point spectra were taken of defined composition mixtures with integration times of 3 x 5 s and a laser intensity of 5 mW. The data points were then fitted with a 3$^{rd}$ order polynomial function so that the concentration of unknown composition can be calculated for the evaporating droplet as it is given in figure 1. From the concentration calculation over the droplet also the concentration gradient (figure 1 (g)) is then calculated.

Since the experimental setup for confocal Raman spectroscopy (as depicted in figure 1(b) in the main manuscript) includes a view from above and the measurements are not taken in the droplet center, the laser always hits non-perpendicularly on the curved droplet surface. This means that the laser radiation is deflected when entering the droplet and that the measuring positions depicted in the 2D Raman scans are shifted to other positions within the droplet. The drop can be handled as a further lens in the optical path of the experimental setup.

In figure S4 this effect that describes the droplet similar to an additional lens in the light path is depicted. The different colors are assigned to different droplet sizes and droplet shapes. The gray dots represent the pixels given in the Raman scan and the colored dots represent the points where the actual measurement was taken and which are then projected onto the gray ones. Each colored dot corresponds to the respective colored droplet line. In the center of the droplet the compression/stretching is predominant, while the distortion of the image can be neglected. In our case, the distortion also can be neglected because of the low resolution of the objective and the small area scanned in the 2D Raman scans compared to the droplet size. The measurement position is also not too far away from the droplet center so that the distortion might not be as dramatic as depicted in figure S4(a,b). Nevertheless, the stretching component of this lens effect was considered and the actual height of the drop was then recalculated. Therefore, a blank 2D Raman scan with only the coated glass substrate was taken. The glass substrate as well as the upper side of the droplet are considered as fixed positions and then this distance was recalculated with the first blank scan as the fixed substrate position.

To prove that the deflection when measuring near the center can be neglected, the focal length of the lens was calculated. For the mathematical derivation a sketch is given in figure S4(c). The figure shows a sketch of the droplet including the mathematical labels, namely the droplet radius ($R$), focal length ($f'$), distance from droplet center ($r$), droplet center ($M$), as well as the angles ($\alpha, \beta, \gamma$) between them. Using those, the focal length is derived. This can then be inserted into the lens grinder formula, from which the refractive power is then calculated, indicating the deflection from the original beam path.

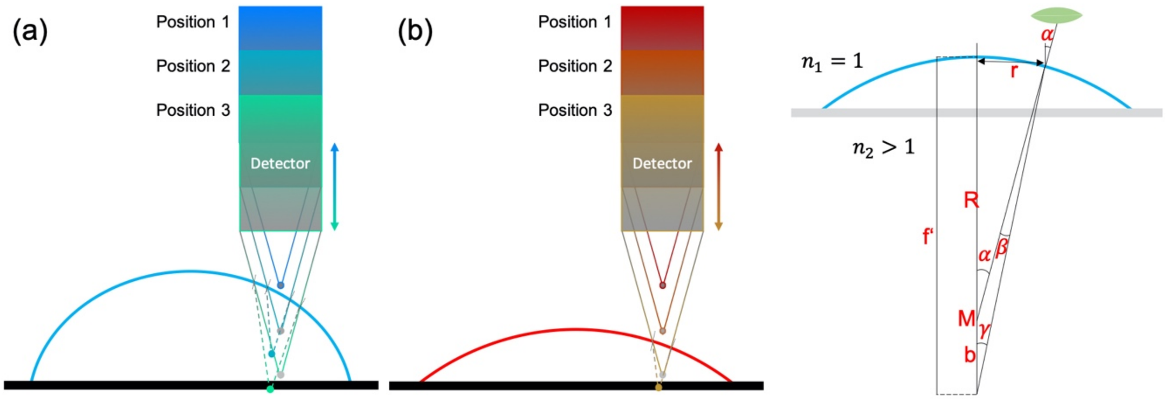


**Figure S4.** Sketch of light refraction on a droplet size in the beginning of the experiments (a) and on a droplet after partial evaporation (b). In the upper part of both images the detector is shown in three different heights from which the measurements were taken. The measurement points are given in the same respective color. The gray dots are the points depicted in the Raman image, the colored dots the ones actually depicted in the gray dots. (c) Sketch of the droplet with mathematical labels to calculate the refractivity of the droplet. The droplet or lens radius is given as *R*, the droplet center *M,* the focal length of the lens *f',* as well as the distance from the droplet center *r*. The angles $\alpha$, $\beta$, and $\gamma$ are also depicted.

The focal length *f'* is calculated adding the radius *R* and the offset *b* due to deflection

$$f' = R + b$$

with

$$b = R \times \frac{\sin\beta}{\sin\gamma}.$$

From Snell's law

$$\sin\beta = \frac{\sin\alpha}{n_2}$$

and the geometrical point of view

$$\sin\alpha = \frac{r}{R}$$

$$\alpha = \beta + \gamma$$

we obtain the following equation:

$$f' = R + \frac{r}{n_2 \times \sin\gamma} = R \times \left(1 + \frac{1}{n_2 \times \cos\beta - \cos\alpha}\right)$$

$$= R \times \left(1 + \frac{1}{\sqrt{n_2^2 + \sin^2\alpha} - \sqrt{1 - \sin^2\alpha}}\right).$$

This leads to

$$f' = R \times \left(1 + \frac{1}{n_2 \times \sqrt{1 - \frac{r^2}{n_2^2 R^2}} - \sqrt{1 - \frac{r^2}{R}}}\right).$$

For small *r* i.e. $\frac{r^2}{R} \ll 1$, this equation can be simplified to

$$f' \cong R \times \frac{n_2}{n_2 - 1}.$$

For large *r* values a Taylor expansion has to be considered which results in the end in

$$f' \approx R \times \left(\frac{n_2}{n_2 - 1} - \frac{r^2}{2n \times (n-1) \times R^2}\right),$$

meaning that for large $r$ the focal distance $f'$ decreases quadratically with the distance from the droplet center. Thus, to reduce geometric optical effects Raman depth scans were taken near the center (small $r$ approximation).
The refractivity ($D$) is given by the lens makers' formula

$$D = \frac{1}{f'}.$$

In case of small $r$ the refractivity is calculated to be inversely proportional to the droplet radius (see following equation) when inserting the focal length calculated before

$$D = \frac{1}{R} \times \left(1 - \frac{1}{n_2}\right).$$

## Non localized NMR spectroscopy of 1-butanol/1-hexanol droplets

Linewidths in NMR spectra depend on the homogeneity of the applied magnetic field. Introduction of (curved liquid air) interfaces disturb the field homogeneity. The impact on the resulting line width depends on the field strength of the spectrometer used. Hence, the impact of the interface of a sessile droplet on the line shape in non localized NMR spectra should be smaller at lower fields. Therefore, we acquired NMR spectra of 1-butanol, 1-hexanol and a 50mol% mixture at 200 MHz proton resonance frequency (4.7 T). As shown in figure S5, the spectral resolution was limited.

Thus, the total concentration of evaporating droplets consisting of 1-butanol/1-hexanol mixtures was determined based on the peak ratio R = S($CH_3$+$CH_2$)/S(O-$CH_2$+OH), where S is the integral of the signal intensity. For this purpose, we integrated the signal intensity over the $CH_3$ + $CH_2$ peaks from 0.1 to 2.85 ppm and the O-$CH_2$ + OH peaks from 2.9 to 6.35 ppm, as shown in figure S5. Thus, the expected ratios for the neat compounds under ideal circumstances were R=7/3 and R=11/3 for 1-butanol and 1-hexanol, respectively. The molar fractions $X_{But}$ and $X_{Hex}$ could be calculated from the R measured at each time point given.

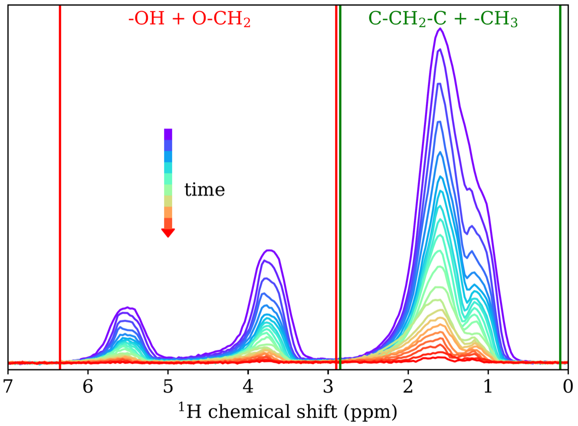


**Figure S5.** $^{1}$H chemical shift spectra of an evaporating droplet of a 1-butanol/1-hexanol mixture on a PFDTS substrate obtained from a non-localized measurement at 200 MHz proton resonance frequency (4.7 T). The initial 1-butanol concentration was 50 mol%, and the initial droplet volume was 4.2 µl. Due to susceptibility artefacts, the spectra are significantly broadened. The vertical lines indicate the integrated areas used to determine the signal intensities of the -OH + O-$CH_2$ peaks (red) and the $CH_2$ + -$CH_3$ peaks (green).

Figure S6 shows the normalized peak ratios for 1-butanol $R_{But}$=R·3/7 and 1-hexanol $R_{Hex}$=R·3/11, which were expected to be constant. To compensate for differences in the evaporation speed, the volume dependence is shown instead of the time dependence. The volume was calculated from the total signal intensity. Deviations of the scaled peak ratios $R_{But}$ and $R_{Hex}$ from unity at larger volumes probably stemmed from water inside the droplets that contributed to the OH peaks. At the end of the evaporation, at volumes below approximately 0.6 µl, the deviations became larger due

to larger effects from magnetic field distortions for smaller droplets. Hence, the experimental results for these small drop volumes were prone to errors and disregarded (see the grey shaded area in figure S6). Exploiting this knowledge, we determined the time-dependent concentrations in the evaporating 1-butanol/1-hexanol mixture (see figure 2).
The molar fractions of 1-butanol ($X_{But}$) and 1-hexanol ($X_{Hex}$) in the evaporating droplet with an initial 50:50 mol% 1-butanol/1-hexanol mixture are shown in figure S6. The concentration of the more volatile component $X_{But}$ decreased during evaporation but did not reach zero. Rather, it appeared to saturate at a concentration of slightly less than 20 mol%. Below that, errors in measurement did not allow further conclusions.

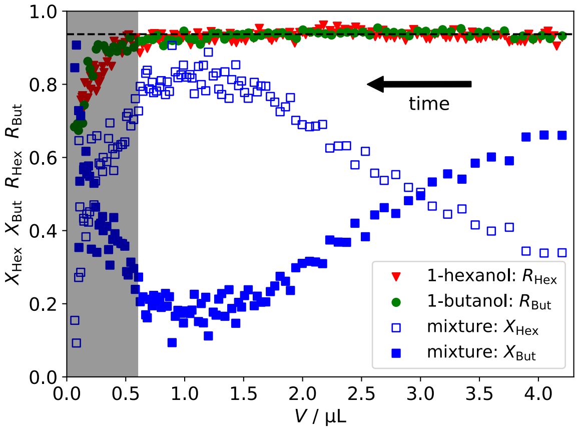


**Figure S6.** Evaporating droplets with initial volumes of 4.2 µl on a PFDTS substrate: 1-butanol, 1-hexanol and a mixture of 1-butanol/1-hexanol with an initial concentration of 50:50 mol%. Green circles and red triangles: Normalized $^1$H peak ratios R = S($CH_3$+C-$CH_2$-C)/S(O-$CH_2$+OH) of 1-butanol $R_{But}$=R·3/7 and 1-hexanol $R_{Hex}$=R·3/11. Horizontal dashed line at 0.937: average of $R_{Hex}$ and $R_{But}$ for V > 2.1 µL. Deviations from 1 probably stem from water inside the droplets. A strong influence from magnetic field distortions appears when the droplet volume is smaller than 0.6 µl (grey shaded area). Molar concentrations of the mixture: $X_{Hex}$ (blue open squares) and $X_{But}$ (blue filled squares).

## <u>Error estimation for spatially resolved NMR Experiments</u>

The error of the 1-butanol and 1-hexanol fraction calculated from PRESS spectra is determined by several properties. In addition to bad signal phases or insufficient baseline correction, which can be avoided by proper processing of the spectra, it is observed that in PRESS spectra a deviation of the relative signal integrals occurs. For pure 1-hexanol a relation of 1:2:8:3 is expected for the integrals of OH, $OCH_2$, $CH_2$, and $CH_3$ resonances. As shown in figure S7 a deviation of ca. 10 % is observed for the integrals of OH and $OCH_2$ resonances. This deviation is also observed in PRESS spectra of the evaporating droplet (figure S8). Hence it is assumed that in mixtures of 1-butanol and 1-hexanol relative signal integral have an inaccuracy of ca. 0.2 so that a minimum error of 5 % is expected.
Regarding the NMR measurements without spatial resolution, the time-dependent spectra of 1-butanol and 1-hexanol are shown in figure S9 (a) and (b). The integration area of the peaks is indicated by the vertical lines and is the same for all three samples. As mentioned and shown in figure S6, the peak ratios amount to $R_{But}$ = R 3/7 for 1-butanol and $R_{Hex}$ = R 3/11 for 1-hexanol. The uncertainty is estimated based on the deviations from these ratios at large volumes and is also about 5%.

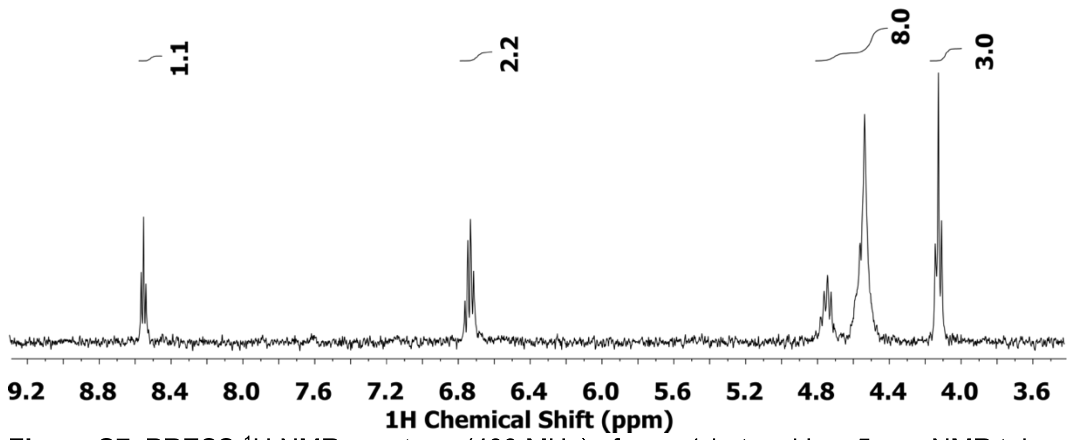


**Figure S7.** PRESS $^1$H NMR spectrum (400 MHz) of pure 1-butanol in a 5 mm NMR tube acquired at 298.5 K in a 0.2 x 0.2 x 0.2 mm voxel. The spectrum was acquired with TE = 20 ms, TR = 4 s, NS = 64 and 8000 points within an acquisition time of 998.4 ms. The total experimental duration was 4 min 35 s.

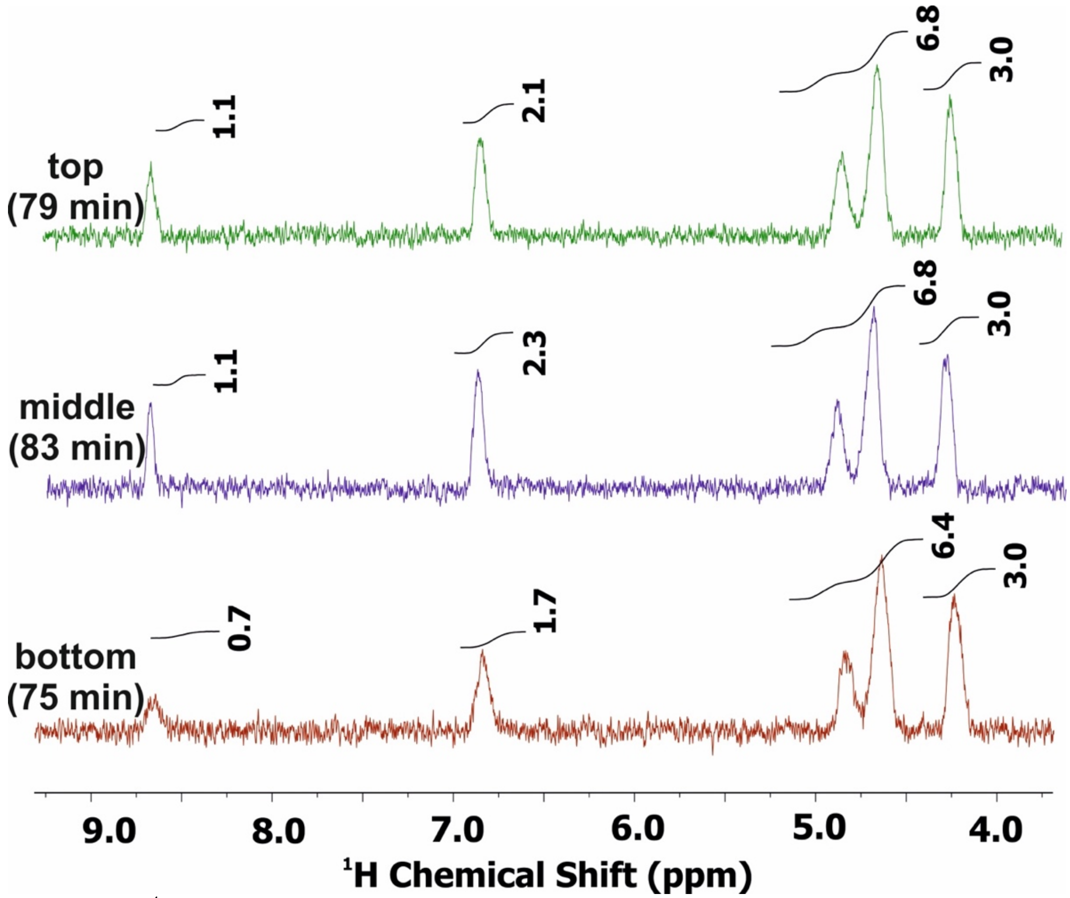


**Figure S8.** $^1$H PRESS NMR spectra (400 MHz) of a 50 mol% mixture 1-butanol and 1-hexanol during evaporation at 298.5 K in a 4.2 µL droplet on a PFDTS surface. As described in the main text the three voxels (0.2x0.2x0.2 mm) are located at different heights of the droplet (red: bottom voxel, after 75 min evaporation; blue: middle voxel, 83 min evaporation and green: top voxel, 79 min evaporation). PRESS spectra are acquired with TE = 20 ms, TR = 4 s, NS = 64 and 8000 points within an acquisition time of 998.4 ms. The total experimental duration of each PRESS experiment is 4 min 35 s. For determination of the 1-butanol fraction the integrals of the $–CH_2$ and $–CH_3$ resonances are compared via equations 1-1 and 1-2.

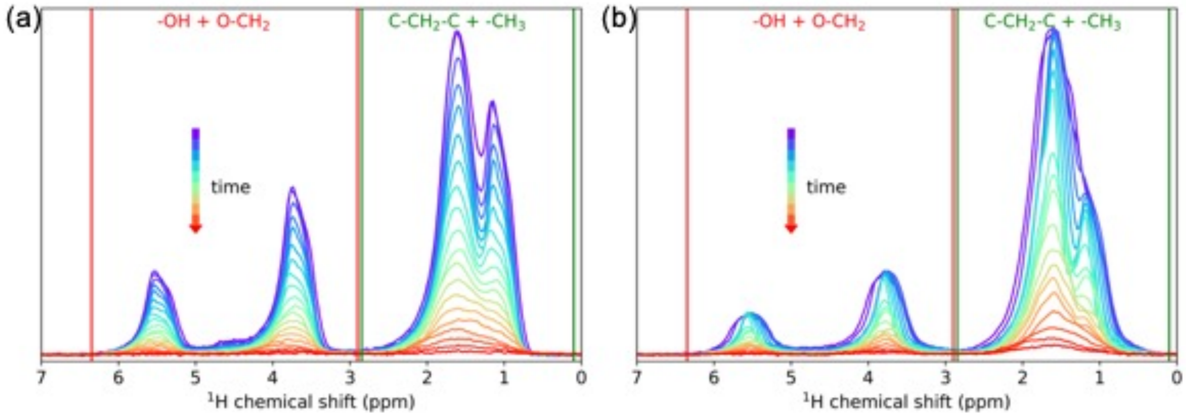


**Figure S9.** $^1H$ chemical shift spectra of an evaporating droplet of (a) 1-butanol and (b) 1-hexanol on a PFDTS substrate obtained from measurements without spatial resolution at 200 MHz proton resonance frequency (4.7 T) according to figure 3 in the main manuscript. The initial droplet volumes were 4.2 µl. Due to susceptibility artifacts the spectra are significantly broadened. Vertical lines indicate the integration areas used to determine the signal intensities of the -OH + O-CH2 peaks (red) and of the CH2 + -CH3 peaks (green), respectively.

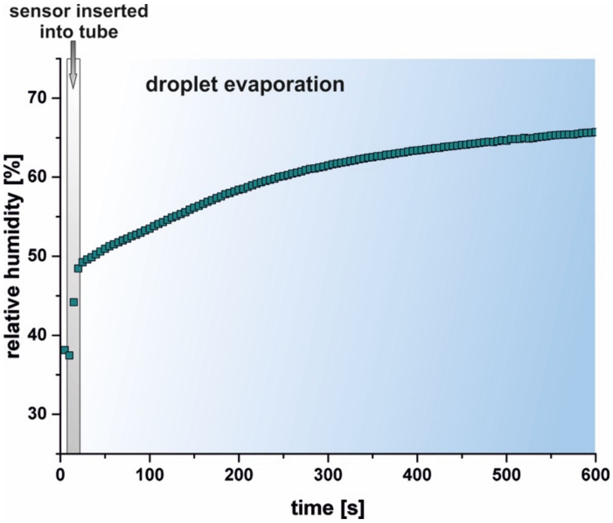


**Figure S10.** Relative humidity inside a 10 mm NMR tube during evaporation of a sessile droplet of water with an initial volume of 4.5 µL on a mesoporous silica surface at room temperature. Prior to insertion of the sensor (Honeywell) into the tube, the sensor measures the relative humidity of the NMR laboratory. During insertion of the sensor the measured relative humidity increases quickly (grey area). Afterwards the humidity inside the tube slowly increases due to the evaporation of the sessile droplet (blue shaded area).

## Flow Field Measurements

Figure S11 shows the evaluated data from the APTV measurements. The fluorescence image is given in (a) with different deformed tracer particles and the marked contact line. A scheme of the droplet with the flow behavior is given in (b) and the measured three-dimensional particle trajectories are given in (c). For better readability, the particle trajectories are shown in 2D in (d) with color coded velocities. The experiments show a very slow Marangoni flow from the droplet center towards the three-phase contact line along the liquid/air interface, see direction of the surface flow in (b). In the bulk and close to the solid substrate the flow is directed towards the droplet center, see marked bulk flow in (b) and also the projection of the trajectories in (c) and (d). Thus, due to the increased evaporation close to the contact line and the Marangoni effect, the surface flow is directed towards the contact line. The reason for the Marangoni flow measurements is the concentration gradient measured with Raman spectroscopy and NMR. In this publication we only discussed the bulk region of the droplet because all Raman spectroscopy and NMR measurements were taken in the bulk of the drop. A detailed analysis of the evaporation dynamics close to the contact line will be discussed in a forthcoming publication.

## Materials and Methods

### Contact Angle Goniometry

Visual measurements of the evaporating droplets were performed in side view mode. The evolution of the droplet shapes over time was recorded with a CMOS camera (UI-1550SE, iDS, Germany) equipped with a long-distance microscope objective (12X, Navitar, USA). Backlight illumination was achieved by scattering the light that left a light guide with a diffusor built in-house. The light source was a cold light source (intraLED5, VOLPI, Switzerland). To achieve repeatable environmental conditions that match those of the Raman measurements, the droplets evaporated inside of a climate chamber built in-house into a humidified atmosphere. The relative humidity was set to 50 +/- 1 % RH by mixing a dry nitrogen stream with a humidified nitrogen stream at the appropriate ratio. The measurements were performed at temperatures between 22.0 °C and 23.5 °C. The acquired image data were evaluated with an in-house software called DENIISE developed by Heinz *et al.*. (8)

The calculation for the droplet radius (R) depended on the wetting radius (r) and CA (θ) as $\mathrm{R} = \frac{\mathrm{r}}{\sin\theta}$. From experiments, the mean values of r and θ, as well as their errors $r_{std}$ and $\theta_{std}$, which were the standard deviations of the respective measurand obtained by rolling mean averaging of the time series extracted by DENIISE, were known. The maximum and minimum radii of curvature $R_{min}$ and $R_{max}$ could therefore be calculated from the experimental data as

$$R_{max} = \frac{r + r_{std}}{sin}$$

and

$$R_{min} = \frac{r - r_{std}}{sin},$$

respectively.

### Flow Field Measurements

Three-dimensional particle trajectories were measured using astigmatism particle tracking velocimetry (APTV). APTV is a single camera technique that uses a planned aberration of the optical system to measure three-dimensional dynamics. (9-12) The setup consists out of an inverted fluorescent microscope (Leica DMI 6000B) and a high-speed camera (Photron Fastcam SA1.1). A cylindrical lens (focal length = 150 mm) is placed in front of the camera chip to create the aberrations. The mercury lamp of the microscope provides illumination. Fluorescent polystyrene particles (2µm, PS-FluoRed: Ex/Em 530nm/607nm, microParticles GmbH) were added to the measurement solution and tracked.

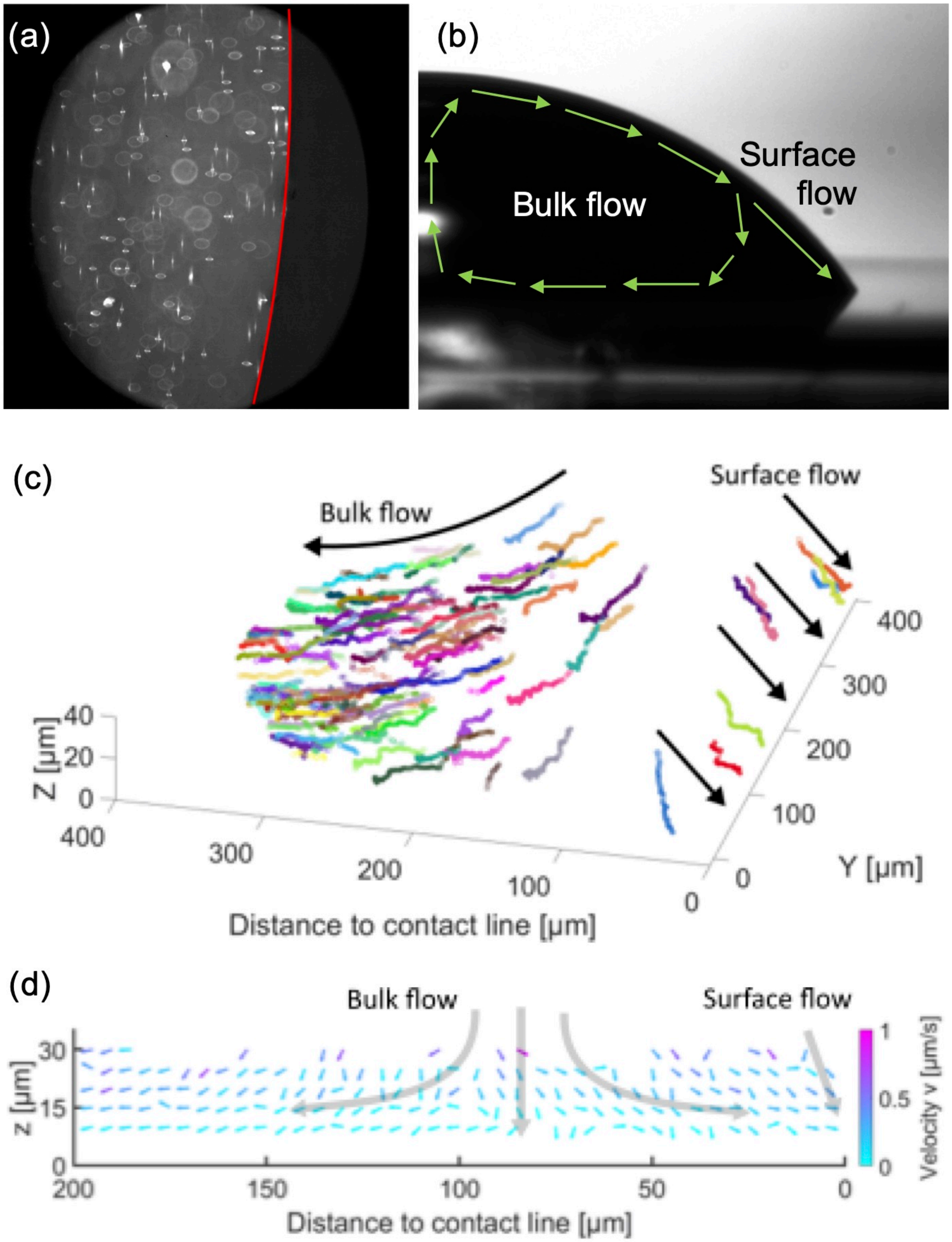


Figure S11. Experimental results from APTV measurements. (a) APTV Fluorescence image of the evaporating droplet with the contact line marked in red. (b) Scheme of the droplet with the flow directions of the bulk flow and the surface flow. (c) Evaluated trajectories in 3D. All trajectories are given in different colors. On the x-axis the distance to the contact line is given (contact line always on right end of the graphics). The flow at the liquid/air interface is directed at the contact line and in the bulk the flow is directed towards the center of the drop. (d) Flow trajectories in 2D with color coded flow velocities.

**Estimation of Flow Regime**

According to Diddens *et al.* convection in evaporating binary sessile droplets can be characterized in three regimes. (13) The first regime is dominated by Marangoni flow the second regime by Rayleigh flow with opposite flow direction. Within the third regime multiple vortices with different flow directions build up within the evaporating droplet. The regimes can be described using a phase diagram using the dimensionless Rayleigh and Marangoni number.

The Rayleigh number describes the ratio of the timescale for thermal transport via diffusion and the time scale for thermal transport via convection and therefor can be written as:

$$Ra = \frac{\Delta\rho \cdot l^3}{\eta \cdot \alpha}$$

With $\Delta\rho$ being the density gradient along the sample, $l$ the dimension, $\eta$ the dynamic viscosity and $\alpha$ the thermal diffusivity.

The thermal diffusivity can be expressed using the thermal conductivity $\lambda$ , the density $\rho$ and the specific heat capacity $c_p$ of the flowing material:

$$\alpha = \frac{\lambda}{\rho \cdot c_p}$$

The Marangoni number describes the ratio of advective transport rate caused by surface tension gradients and diffusive transport rate and can be written as:

$$Ma = \frac{\Delta\gamma \cdot L}{\eta \cdot D}$$

For binary butanol/hexanol and butanol-$d_9$/hexanol droplets the flow regime is estimated here using the following values shown in table S1. For the system 1-butanol/1-hexanol a concentration gradient of 10 mol% is observed at the beginning of the evaporation. Assuming that the density scales linearly with composition the difference in densities $\Delta\rho$ within the droplet is at the order of 0.4 kg/m³. Using the droplet volume of ~ 3.2 µL as volume $l^3$ a Rayleigh number of approximately 104 is obtained. For the system 1-butanol-$d_9$/1-hexanol the difference in densities is larger due to the higher density of 1-butanol-$d_9$. Here a $\Delta\rho$ of 9.3 kg/m³ is obtained and the Rayleigh number increases to approximately 2400.
For estimation of the Marangoni number, it is assumed that the surface tension gradient $\Delta\gamma$ can be approximated by the difference in surface tension due to the local differences in concentrations and that the surface tension scales linearly with composition. For a 1-butanol/1-hexanol system with a concentration gradient of up to 10 mol% the surface tension gradient is approximately 0.0032 N/m³. With that a maragoni number of approximately 10000 is obtained.
According to Diddens *et al.* (13) for both droplets (with contact angle of approx.. 50°) Marangoni flow should dominate the flow inside the droplet, thus one would expect convection within the droplet.

## SI References


1. L. Andreoli-Ball, D. Patterson, M. Costas, M. Cáceres-Alonso, Heat capacity and corresponding states in alkan-1-ol–n-alkane systems. *Journal of the Chemical Society, Faraday Transactions 1: Physical Chemistry in Condensed Phases* **84**, 3991-4012 (1988).
2. W. Haynes (2014) CRC Handbook of Chemistry and Physics 94th Edition Internet Version.
3. X. Chen, R. Hu, H. Feng, L. Chen, H.-D. Lüdemann, Intradiffusion, Density, and Viscosity Studies in Binary Liquid Systems of Acetylacetone + Alkanols at 303.15 K. *Journal of Chemical & Engineering Data* **57**, 2401-2408 (2012).
4. C. Y. Wang, M. L. Yang, A new calorimeter for measuring rapidly the thermal conductivity of liquids. *Thermochimica acta* **255**, 365-370 (1995).
5. T. Atrashenok, N. Nesterov, I. Zhuk, A. Peshchenko, Measured specific heats of hexan-1-ol and 3-methyl-2-butanol over wide temperature ranges. *Journal of engineering physics* **61**, 1038-1041 (1991).
6. M. Iwahashi *et al.*, The dynamical structure of normal alcohols in their liquids as determined by the viscosity and self-diffusion measurements. *Bulletin of the Chemical Society of Japan* **59**, 3771-3774 (1986).
7. J. K. Park, J. Ryu, B. C. Koo, S. Lee, K. H. Kang, How the change of contact angle occurs for an evaporating droplet: effect of impurity and attached water films. *Soft Matter* **8**, 11889-11896 (2012).
8. M. Heinz, P. Stephan, T. Gambaryan-Roisman, Influence of nanofiber coating thickness and drop volume on spreading, imbibition, and evaporation. *Colloids and Surfaces A: Physicochemical and Engineering Aspects* https://doi.org/10.1016/j.colsurfa.2021.127450, 127450 (2021).
9. H. P. Kao, A. S. Verkman, Tracking of single fluorescent particles in three dimensions: use of cylindrical optics to encode particle position. *Biophysical Journal* **67**, 1291-1300 (1994).
10. C. Cierpka, M. Rossi, R. Segura, C. J. Kähler, On the calibration of astigmatism particle tracking velocimetry for microflows. *Measurement Science and Technology* **22**, 015401 (2010).
11. M. Rossi, C. J. Kähler, Optimization of astigmatic particle tracking velocimeters. *Experiments in Fluids* **55**, 1809 (2014).
12. B. B. Straub *et al.*, Flow profiles near receding three-phase contact lines: influence of surfactants. *Soft matter* **17**, 10090-10100 (2021).
13. C. Diddens, Y. Li, D. Lohse, Competing Marangoni and Rayleigh convection in evaporating binary droplets. *Journal of Fluid Mechanics* **914**, A23 (2021).
